# Supplementary material for: Coastal sedimentation across North America doubled in the 20th century despite river dams
Source: Nat Commun. 2020 Jun 26;11:3249. doi: 10.1038/s41467-020-16994-z (PMC7319974; doi:10.1038/s41467-020-16994-z)
Supplement: Supplementary file 1 — Supplementary Information [file 41467_2020_16994_MOESM1_ESM.pdf]

# Supplementary Information for:

# Coastal sedimentation across North America doubled in the 20th century despite river dams

**Rodriguez, et al., Nature Communications**

**Supplementary Note 1**

Additional sedimentological and geochemical information about the cores used in this study are summarized from the original published work, below. See Supplementary Data 1 and Supplementary Data 2 for more details.

Sedimentological and geochemical data from Nastapoka Sound, Canada is from Jolviel et al. (2015)[1]. Core C13 is included in our study and that core was collected ~7 km northwest of the Sheldrake River outlet in 2009 and is 38 cm in length (labeled Site 1 in Fig. 1). A CT-Scan of the core at 1 mm resolution did not image any sedimentary structures, ice rafted debris, or burrows. The core was sectioned at 1-cm intervals. Grain-size analyses using a 500 µm sieve for the coarse fraction and a Horiba laser sizer for the fine fraction shows that the core sections with excess Pb-210 are composed of coarse silt with a mean grain-size range of 29-35 µm. Organic carbon was measured using a Carlo-Erba elemental analyzer and ranges between 0.43% to 0.54% in the upper 7 cm of the core. The Cs-137 peak was identified between 3- and 4-cm depth in the core. The depth of supported Pb-210 levels was 7 cm from the top of the core. Core C13 was one of 6 cores selected for the study and reported average sedimentation rates based on the "constant flux-constant sedimentation" model ranged between 0.13 and 0.02 cm $yr^{-1}$ with a median of 0.07 cm $yr^{-1}$. The reported average sedimentation rate for Core C13 is just below the median, or 0.06 cm $yr^{-1}$. We chose to include Core C13 because it was collected from the center of the depocenter and contained the longest record.

Lima et al. (2005)[2] collected a 165-cm long core from the deepest part of the lower Pettaquamscutt River Basin, Rhode Island, USA in 1999 (labeled Site 2 in Fig. 1). The basin contains anoxic bottom water due to stable salinity stratification of the water column. X-radiographs of the core show laminated sediments (annual varves) and no burrows. The core was section at a 1-cm interval from 0-1 cm and 2.0-cm interval from 1-165 cm. The organic carbon content of subsamples was measured using a Fisons 1108 elemental analyzer and ranged between 8.1% at the surface to 9.8% below 30-cm depth in the core. The seasonal laminae were composed of a spring/summer-deposited layer of predominately fine-grained organic material and diatom frustules and a fall/winter-deposited layer of predominately fine-grained clastic material. Fall/winter laminae of fine sand associated with hurricanes in 1954 and 1938 were also recognized. A well-defined Cs-137 peak was identified in the 18-18.5 cm depth sample. Pb-210 chronologies were confirmed by varve counting, the Cs-137 peak, and radiocarbon dating (post-bomb calibration). The depth of supported Pb-210 levels was 44.25 cm from the top of the core. More than one core was obtained from the site (unclear exactly how many) but the authors report that each core showed the same number of laminae.

Renfro et al. (2016)[3] collected Core 2 (32-cm long) from the southeastern part of Jamaica Bay, New York, USA in 2008 (labeled Site 3 in Fig. 1). The upper 20 cm of the core was sectioned into 2-cm intervals and 4-cm intervals between 20-32-cm depth. No changes in sediment texture downcore were noted, based on the percent sand of subsamples determined by wet sieving through a 63 µm mesh. Organic content was measured using loss on ignition and except for an outlier at 6-8 cm (14%), values were between 6% and 7%. The Cs-137 profile shows a peak between 20-22-cm depth in the core, and profiles of pharmaceutical compounds in

the cores are consistent with known chronologies of their use in the USA, which is interpreted to indicate bioturbation is insignificant. The depth of supported Pb-210 levels was 30 cm from the top of the core. Core 2 was one of 8 cores collected in the study and reported average sedimentation rates based on the “constant initial activity” model range between 1.1 and 0.1 cm $yr^{-1}$ with a median of 0.85 cm $yr^{-1}$. Core 2 has the second lowest reported average sedimentation rate (0.5 cm $yr^{-1}$) but was chosen because it was from a deep muddy part of the bay away from the most developed shorelines.

Benninger et al. (1981)[4] collected Core BX6 from the landward extension of the Hudson Shelf Valley in 1975, the principal focal area of fine-grained sediment accumulation in the inner New York Bight, USA (labeled Site 4 in Fig. 1). The 39-cm long core was composed of brown to black organic-rich silt and clay sampled at irregular intervals including 0-1 cm, 2-4 cm, 4-7 cm, 9-11 cm, 14-17 cm, 21-24 cm, 28-31 cm, and 36-39 cm. Based on water content alone, the authors interpreted a gradual transition from clay and silt (mud) in the upper 10 cm of the core to sandy mud at the base of the Pb-210 profile (a decrease in water content of 29%). X-radiographs of the core show little structure and “evidence of bioturbation is meagre.” Organic matter was measured using loss on ignition and decreases down core from 11.4% at the top to 5.0 % at 21-24 cm. The Pu-239, 240 profile shows relatively high values from the surface to 17-cm depth in the core (217-299 dpm $kg^{-1}$ dry mass) and sharply decreases to 6.7 dpm $kg^{-1}$ dry mass at the bottom of the core. The depth of supported Pb-210 levels was 36 cm from the top of the core. Core BX6 was one of 5 cores included in the study and reported average sediment accumulation rates based on the “constant flux-constant sedimentation” model ranged between 0.36 and 0.51 g $cm^{-2}$ $yr^{-1}$ with a median of 0.41 g $cm^{-2}$ $yr^{-1}$. Core BX6 was below the median with a reported value of 0.38 g $cm^{-2}$ $yr^{-1}$. We chose BX6 because it was located at the head of the canyon.

Holmes and Marot (2004)[5] collected Core 6B 3-km south of Watts Island, about 30 km southwest of the Pocomoke River outlet, Virginia, USA, in 2001 (labeled Site 5 in Fig. 1). The 346-cm long core was subsampled in 2-cm increments. X-radiographs of the core show distinct horizontal bedding (1 to 10-cm thick). Sediment texture was determined using a Beckman Coulter LS200 Particle Analyzer and was mostly silty clay. The authors noted that “there is an indication that the post 1950 sediment was slightly coarser.” Organic matter content of the core was measured from loss on ignition and shows no trend with depth and ranged from 4 to 8 %. The Cs-137 activity shows a distinct peak at around 50-cm depth in the core. The authors noted an increase in anthropogenic elements and a decrease in lithic elements in the subsamples around AD 1950 and noted that “urbanization and agricultural activity changed during and immediately following World War II resulting in increasing sediment flux.” The depth of supported Pb-210 levels was 60 cm from the top of the core. Core 6B was one of 6 cores included in the study and reported average sediment accumulation rates based on the “constant flux-constant sedimentation” model ranged between 0.25 and 1.8 cm $yr^{-1}$ with a median of 0.47 cm $yr^{-1}$. Core 6B was reported to have the highest average rate but the authors noted that cores 2, 3, 4, and 6 all showed an abrupt increase in sediment accumulation rate between 1953 ± 4 yr and 1959 ± 4 yr. Core 6B was chosen because it was collected from the center of the basin where the sedimentary record was longest.

Corbett et al. (2007)[6] collected core S1 from the center of Albemarle Sound, North Carolina, USA in 2001, about 15 km east of the Roanoke Bayhead Delta and in the western half of the sound (labeled Site 6 in Fig. 1). The 40-cm long core was extruded into 2-cm intervals to 4 cm depth and 3-cm intervals from 4 cm to the base. Sediment texture of the subsamples was measured using a Beckman Coulter LS230 Particle-size Analyzer and shows little variation down core (subsamples contained between 1% and 3% sand). A well-defined Cs-137 peak was recognized at 20 cm depth in the core. Percent organic matter, determined from loss on ignition, increases from about 7.5% at the limit of Pb-210 supported levels at 30 cm core depth to 11 % at the top of the core. Core S1 was one of 4 cores collected along the east-west axis of Albemarle Sound (65 km-long transect) and reported average sediment accumulation rates based on the "constant flux-constant sedimentation" model ranged between 0.57 and 0.08 cm $yr^{-1}$ with a median of 0.16 cm $yr^{-1}$. Those average sediment accumulation rates decrease away from the Roanoke Bayhead Delta. Core S1 was reported to have the highest average rate but was chosen because it was the only core with more than 5 subsamples.

Cooper et al. (2004)[7] collected Core M4 from the center of the Pamlico River Estuary, North Carolina, USA near Gum Point and ~30 km southeast of the Tar River outlet in 1997 (labeled Site 7 in Fig. 1). An X-radiograph of the 137-cm long core revealed uniform stratigraphy; no evidence of sand layers, and no areas of mixing or bioturbation. The core was extruded and sectioned every 1-2 cm. The sediment consisted of clay and silt with small sand particles. The Cs-137 activity showed a clear peak at 40 cm depth. Sediment organic matter was measured using loss on ignition and shows a gradual decrease down core from 5% at 2 cm depth to 3% at the bottom of the core at 120-cm depth. The authors note an increase in SAR at 1950 and attribute that increase to land-use change. The depth of supported Pb-210 levels was 64 cm from the top of the core. Core M4 was one of 3 cores collected in Pamlico Sound and reported average sediment accumulation rates based on the "constant rate of supply" model were calculated from 1947-1997 and 1850-1947 for each core. All cores show an increase in sediment accumulation rate through time. Sediment accumulation rates for cores BC, M4, and BB were 1.20, 1.15, and 0.27 cm $yr^{-1}$ from 1947 to 1997 and were 0.12, 0.26, and 0.11 cm $yr^{-1}$ from 1850 to 1947, respectively. Raw data were only presented for Core M4.

Benninger and Wells (1993)[8] collected Core NRE from the center of the Neuse River Estuary, North Carolina, USA, ~22 km southeast of the Neuse River outlet, and in the western half of the Estuary in 1982 (labeled Site 8 in Fig. 1). The 40-cm long core was sectioned into 2-cm intervals and is composed of silt and clay particles. A well-defined Cs-137 peak was recognized at a depth of 10-12 cm below the top of the core. The depth of supported Pb-210 levels was 34 cm from the top of the core. Multiple cores (10) were collected in this study but average sediment accumulation rates were not reported. We chose core NRE because it was collected from the center of the estuary and contained one of the longest records with a well-defined Cs-137 peak. Cooper et al., 2004 reported average sediment accumulation rates based on the "constant rate of supply" model from 1947-1997 and 1850-1947 for 3 cores in the Neuse River Estuary (in addition to the Pamlico Sound cores reported above). All cores show an increase in sediment accumulation rate through time. Sediment accumulation rates for cores OR,

WP, and UNC were 0.60, 0.61, and 0.22 cm $yr^{-1}$ from 1947 to 1997 and were 0.20, 0.28, and 0.16 cm $yr^{-1}$ from 1850 to 1947, respectively.

Wachnicka et al. (2013)[9] collected the Russell Bank core from the middle of Florida Bay, Florida, USA near Russell Key in 2002 (labeled Site 9 in Fig. 1). X-radiographs of the 167-cm long core revealed "some evidence of bioturbation or disruption of sedimentary layers". The core was sectioned into 2-cm intervals and was composed of medium to light grey, fine-grained carbonate mud interspersed with shell and plant "layers". The sediments are biological and accumulation rates were interpreted to vary with salinity, which is controlled by river and wetland management. The depth of supported Pb-210 levels was 97 cm from the top of the core. The Russell Bank Core was one of 4 cores collected in Florida Bay and reported average sediment accumulation rates based on a "secular equilibrium model in which all data points are used in the sediment accumulation rate calculation" were 0.4 cm $yr^{-1}$ for the Bob Allen Bank core, 0.5 cm $yr^{-1}$ for the Ninemile Bank core, 0.6 cm $yr^{-1}$ for the Trout Cove core, and 1.1 cm $yr^{-1}$ for the Russell Bank core. The Trout Cove and Bob Allen cores showed evidence of bioturbation and reworking. We chose to include the more complete Russell Bank Core over the Trout Cove core for our analysis. Holmes et al. (2001)[10] collected three cores from Russel Bank in 1995, RB 19A, RB 19B, and RB 19C within 54 m of each other. Average sediment accumulation rates, based on Pb-210 profiles, of 1.22 cm $yr^{-1}$ were measured on both cores RB 19A and RB 19C (core RB 19B was collected adjacent to RB19A and was not analyzed). Orem et al., (1999)[11] collected core FB-2 from Russell Bank in 1996 and based on the Pb-210 profile and the "constant rate of supply" model measured the average sedimentation rate as 0.93 cm $yr^{-1}$. Those previous studies support the results of Wachnicka et al. (2013)[9].

Smith and Osterman (2014)[12] collected Core 8BC in 2010 from southern Mobile Bay, Alabama, USA, ~10 km northeast of Main Pass tidal inlet outside the area where dredged material is disposed (labeled Site 10 in Fig. 1). Sediment remobilization primarily takes place in the middle of Mobile Bay and core 8BC is likely a sediment focusing area. The 35-cm long core was sectioned into 1-cm intervals. The Cs-137 profile shows no distinguishable peak; however, the first detection of the isotope at 10-cm depth was apparent. Sedimentation rates based on the first occurrence of Cs-137 match Pb-210 derived values. On average, 90% of the core was composed of silt and clay. There is an increase in sand to about 30% around 7-9 cm depth in the core. Hurricanes Camille and/or Frederic could be the source of sand. Sand content decreases again towards the top of the core (upper 15 cm). Total organic carbon (TOC) measured using a PDZ Europa ANCA-GSL elemental analyzer on subsamples decreases gradually down core from about 24 to 12 mg/g. The depth of supported Pb-210 levels was 34 cm from the top of the core. Core 8BC was one of 3 cores collected in lower Mobile Bay and reported average sediment accumulation rates based on the "constant flux-constant sedimentation" model was 1.63 cm $yr^{-1}$ for 7BC, 0.51 cm $yr^{-1}$ for 8BC, and 0.38 cm $yr^{-1}$ for 20BC. Those three cores are equally spaced along a 20 km-long east-west transect with7BC being the most eastern core. We chose core 8BC because it contained the longest record from the lower part of the estuary.

Draut et al. (2005)[13] collected core OI in 2001 about 125 km west of the Atchafalaya River outlet, Louisiana, USA, from the inner shelf (labeled Site 15 in Fig. 1). The 210-cm long

core was sampled at 5 cm intervals with each sample containing 2 cm of vertical thickness. The sand fraction of the subsamples was separated using a 63 μm sieve and the silt–clay fraction was analyzed using a Micromeritics SediGraph 5100 particle-size analyzer. The upper 170 cm of the core was composed of clay and silt with < 1% sand, interpreted as prodelta, overlaying a stiff shelly sandy mud interpreted as a pre-deltaic inner shelf unit. Excess Pb-210 was only present in the upper 170 cm of the core (the river-dominated shelf sediment) and X-radiographs of that upper unit show it to be homogeneous, interpreted to represent heavy bioturbation. A Cs-137 peak, however, was detected around 115-cm depth in the core. The depth of supported Pb-210 levels was 170 cm from the top of the core. Core OI was one of five cores collected for the study, but only cores OI and OF sampled the river-dominated shelf. Accumulation rates were derived using the Constant Rate of Supply Model and century-averaged accumulation rates for cores OF and OI were reported as 0.94 cm $yr^{-1}$ and 2.0 cm $yr^{-1}$, respectively. Core OI was chosen for this study because Core OF only had 4 samples with excess Pb-210 below the surface mixed layer.

Ravichandran et al. (1995)[14] collected Core St7 ~ 20 km south of the of the Sabine and Neches river outlets from Sabine Lake, Texas, USA, in 1993 (labeled Site 16 in Fig. 1). The 50-cm long core was sectioned into 1-cm intervals from 0-10 cm, 2-cm intervals from 10-20 cm and 3-cm intervals from 20-50 cm depth in the core. The subsamples were separated into mud (silt + clay) and sand fractions using a 63 μm sieve. The authors measured a distinct peak of Pu-239, 240 activity at 12 cm and interpret the profile of Pu to represent small mixing rates from 0-15 cm depth, and a possible increase in mixing rates from 15-35 cm. The depth of supported Pb-210 levels was 24 cm from the top of the core. Core St7 was one of four cores collected for the study. One core was obtained from the upper estuary near the outlets of the Sabine and Neches rivers, two cores were obtained from the middle estuary and core St7 was obtained from the lower estuary. Accumulation rates were derived using the Constant Rate of Supply Model and reported average sediment accumulation rates for the upper estuary core was 1.39 cm $yr^{-1}$, for the middle estuary cores was 0.68 and 0.47 cm $yr^{-1}$ and core St7 from the lower estuary was 0.90 cm $yr^{-1}$. Core St7 was chosen because it was shown to have the least amount of mixing and the most complete sedimentary record.

Santschi et al. (2001)[15] collected the core about 20 km southwest of the Trinity River outlet from Trinity Bay in 1995, which is commonly referred to as being part of Galveston Bay, Texas, USA (labeled Site 17 in Fig. 1). The 44-cm long core was sectioned into 1-cm intervals. Using downcore variations in water and Al content as a proxy for grain size variations, the authors note that grain size variations are relatively small throughout the core (variations were less than 20%) and they measured a Pu-239, 240 peak 10-14-cm depth in the core. The depth of supported Pb-210 levels was 18 cm from the top of the core. An average accumulation rate of 0.29 cm $yr^{-1}$ was derived using the Constant Initial Concentration Model. This was the only core from Galveston Bay collected for the study.

Yaeger et al. (2006)[16] collected Core 4 from the center of the estuary ~18 km from the confluence of Nueces and Corpus Christi bays, Texas, USA, in 2002 (labeled Site 18 in Fig. 1). The 30-cm long core was sectioned into 1-cm intervals and the core was dominantly composed

of clay based on grain-size analyses using a 63 μm sieve for the coarse-grained fraction and a hydrometer for the fine-grained fraction. A peak in Cs-137 was recognized at 8-cm depth from the top of the core. The depth of supported Pb-210 levels was 20 cm from the top of the core. Cores were collected at five sites in Corpus Christi Bay in a transect from the confluence with Nueces Bay towards Mustang Island. Average mass accumulation rates based on the constant flux model were reported to range between 0.18 and 0.30 (median 0.22) g $cm^{-2}$ $yr^{-1}$ with Core 4 being 0.22 g $cm^{-2}$ $yr^{-1}$.

Diaz-Asencio et al. (2009)[17] collected the core ~2 km north of the Sagua la Grande River outlet, Cuba, in 2005 (labeled Site 19 in Fig. 1). The 100-cm long core was sectioned into 1.5-cm intervals. The grain size of each sample was estimated using the gravimetric method after removing gravel-size material (shell) with a 2 mm sieve. The authors described the core as being composed of homogeneous clay and silt-clay with the total clay fraction ranging from 70-95%. The metals Al and Ti were measured by X-ray fluorescence with a SPECTOR X-LAB PRO 2000 system and were distributed uniformly along the length of the core, interpreted by the authors to indicate the same mineralogical origin of the sediments throughout the depositional record. The Cs-137 vertical profile showed a clear maximum at 22.5 cm depth in the core. The depth of supported Pb-210 levels was 48 cm from the top of the core. Only one core was collected for this study.

Alonso-Hernandez et al. (2006)[18] collected Core S1999 from the southern part of Cienfuegos Bay, Cuba, ~3 km southwest and northwest of the outlets of the Caonoa and Arimao rivers, respectively, in 1999 (labeled Site 20 in Fig. 1). The 100-cm long core was sectioned into 1.5-cm intervals. The authors used a Micrometrics Sedigraph 5000ET to measure grain-size of the < 2 mm fraction and used X-ray diffraction to analyze the mineralogical composition of the samples. The core is uniformly clay (70-95% clay) with the median grain size ranging from 6.15 to 4.74 μm. The authors noted that the mineralogical composition of the core reflected that of the surface sediments in the same area and observed no significant variation in the vertical profile. The Cs-137 profile shows 2 small peaks above the initial largest peak at 29.5 cm depth in the core, interpreted to represent pulses of rapid sedimentation from deforestation of adjacent watersheds. The depth of supported Pb-210 levels was 50 cm from the top of the core. Cores were collected at 3 sites in Cienfuegos Bay, two cores from the northern estuary near the river outlet (N1999 and N2000) and one core in the southern part of the estuary (S1999). The three cores show a similar history of sedimentation as modeled using the Constant Flux Constant Sedimentation (CFCS) model, but we chose the southern core because it was less influenced by precipitation events.

> "In the first half of 1900, sediments were regularly accumulated both in the northern and southern basins, at a rate around 0.3 g $cm^{-2}$ $yr^{-1}$. In the following period, the radionuclides vertical profiles significantly changed. In recent years, the sediment accumulation rate almost doubled in the northern basin and the incidence of extreme meteorological events is marked by the deposition of uniform thick sediment layers. The northern rivers have assumed a dominant role in the bay and, after 1963, they have started to supply fine particles also to the

southern basin, as marked by the appearing of chlorite in the southern core. All environmental changes are strictly correlated to substantial changes in land use occurred in the area in the early sixties…"[18]

Ruiz-Fernandez et al. (2009)[19] collected core TEHUA II-21 in the Gulf of Tehuantepec, Mexico, ~24 km from the Tehuantepec River outlet in 2004 (labeled Site 21 in Fig. 1). The 18-cm long core was sectioned into 0.3-cm intervals down to 10 cm and 1-cm intervals from 10 cm to 18 cm. No evidence of bioturbation was found during subsampling. Grain size was determined by sieving and pipetting analysis, the carbonate content of the sediment was measured by acid digestion, and the total carbon content of the sediment was measured using a Carlo Erba™ NC2500 elemental analyzer. The core was mostly composed of sand (76.1-89.0%) with low organic carbon (< 0.8%). Carbonate varied little throughout the core from 12-25%. A well-defined Cs-137 peak was identified at 6.75-cm depth in the core. The depth of supported Pb-210 levels was 15 cm from the top of the core. Only one core was collected from the site because the authors had difficulty retaining sediment in the core liner. Both the Constant Flux Constant Sedimentation and the Constant Rate of Supply models were used, and the authors interpreted changes in the sedimentary record similarly to our study.

"The chronological variations of the sedimentation rates in core Tehua II-21 followed a similar pattern to the population growth in the coastal zone around Salina Cruz harbor (Fig. 5). In fact, from the slopes of the linear regression of each profile it is shown that both rates increased at a slower pace before the 1950s (0.002 cm $yr^{-1}$ per year and 153 inhabitants $yr^{-1}$, respectively) than after the 1980s (0.004 cm $yr^{-1}$ per year and 1521 inhabitants $yr^{-1}$, correspondingly)."[19]

Ruiz-Fernandez et al. (2002)[20] collected core RC98 ~17.5 km upstream from the Culiacan River terminus from a permanently flooded hypo-saline section of the Culiacan River Estuary, Mexico, in 1998 (labeled Site 22 in Fig. 1). The 35-cm long core was sectioned into 1-cm intervals and analyzed for grain size using sieves and a pipette and carbonate content was measured by acid digestion. The sediments were composed mainly of sand (> 90%) with carbonate content ranging from 0.1 to 3.1%. Cs-137 activities in the core did not exceed the analytical background levels and this was interpreted as the result of desorption as the sediment contacted seawater. Analyses of Mn, Al and Fe were made by atomic absorption spectrophotometry and the Fe and Al profiles varied little, which was interpreted to indicate that the sediments came from the same detrital source. The depth of supported Pb-210 levels was 19 cm from the top of the core. One core was collected from the site and the Constant Rate of Supply dating model was applied. Sedimentation rates varied from 0.04 to 1.2 cm $yr^{-1}$; and mass accumulation rates from 0.04 to 1.7 g $cm^{-2}$ $year^{-1}$, "showing the highest value and the most abrupt change around the early 1950s"[20].

Alexander and Lee (2009)[21] collected core S0304SC-5 ~10 km seaward of the shoreline and ~15 km west of the Santa Clara River outlet, California, USA, in 2004 (labeled Site 23 in Fig. 1). The 42-cm long core was sectioned into 1-cm intervals in the upper 10 cm and 2-cm intervals from 10 cm to the base. X-radiographs were obtained and do not show extensive modification of the seabed. Grain size was measured using a combined sieve (for the sand

fraction) and SediGraph 5100 (for the silt and clay fraction) method. The core was composed of muddy sediment and the base of Cs-137 activity was at 20 cm depth in the core. The depth of supported Pb-210 levels was 35 cm from the top of the core. A total of 13 box cores were collected from the Santa Clara Shelf. Mass accumulation rates and sediment accumulation rates were derived using the Constant Initial Concentration model (SAR max=0.99, min=0.26, and median=0.68 cm $yr^{-1}$; MAR max=1.35, min=0.35, and median=0.7 g $cm^{-2}$ $yr^{-1}$). Sedimentation generally decreased away from the shoreline. Core S0304SC-5 was the most distal core and had the lowest SAR and MAR values. Core S0304SC-5 was chosen for our study because it was the only core from the site that sampled a complete Pb-210 inventory.

Fuller et al. (1999)[22] collected core RB92-3 ~1 km from the shoreline and near the mouth of Richardson Bay, a small embayment located 5.6 km northeast of the mouth of San Francisco Bay, California, USA, in 1992 (labeled Site 24 in Fig. 1). The 140-cm long core was subsampled at 4-cm depth intervals for radioisotope analyses. Grain-size analysis was performed on samples of 1-cm width at 10-cm intervals but the method used was not indicated. The X-radiograph of the core showed a few faint laminations at 20-23 cm and 59-60 cm depth in the core. The core contains scattered shells below 50 cm and a relatively constant grain size in the upper 60 cm (~60% mud), with a higher sand content below 60 cm (~50%). Burrows were observed at 3-8 cm, 39-42 cm, 48-50 cm, and 99-101 cm depth in the core. The profiles of Cs-137 and Pu 239, 240 have broad maxima between 33 and 41 cm depth in the core with detection limits reached at the 53-57 cm depth interval. The depth of supported Pb-210 levels was 65 cm from the top of the core. At the mouth of Richardson Bay, 8 stations were sampled by collecting one 0.5 m-long box core, two 2-m long gravity cores, and one 2-m long freeze core within 9 m of each other. Average mass accumulation rates were calculated by applying the Constant Flux Constant Sedimentation model (0.825 g $cm^{-2}$ $yr^{-1}$ for core RB92-3). Richardson Bay data were only provided for core RB92-3. The gravity core was chosen because it contained a more complete record than the box core but Pb-210 profiles were similar.

Wheatcroft and Sommerfield (2005)[23] collected core CHT 15 km northwest of the Chetco River outlet on the continental shelf offshore of Oregon, USA, in 1998 (labeled Site 25 in Fig. 1). The core was composed of mud and there was no evidence of drastic down-core grain size changes and X-radiographs show no evidence of physical bedding or flood layers. The method used for grain-size analysis was not indicated. The 48-cm long core was sectioned into 1-cm intervals. A broad peak in the Cs-137 profile was measured between 10-20 cm and the detection limit was reached at 40 cm depth in the core. The depth of supported Pb-210 levels was 49 cm from the top of the core. This paper reported on 24 Pb-210 profiles offshore of the Umpqua, Rogue, Chetco, Smith, Klamath, and Russian rivers. Only one core was collected offshore if the Chetco River. Sediment accumulation rates were derived using the Constant Initial Concentration dating model and were 5.8 mm $yr^{-1}$. A core collected offshore of the adjacent Smith River (to the south) had an average SAR of 5.7 mm $yr^{-1}$. We chose the more complete Chetco River core over the other sites for this study, which also had the least evidence of surface reworking.

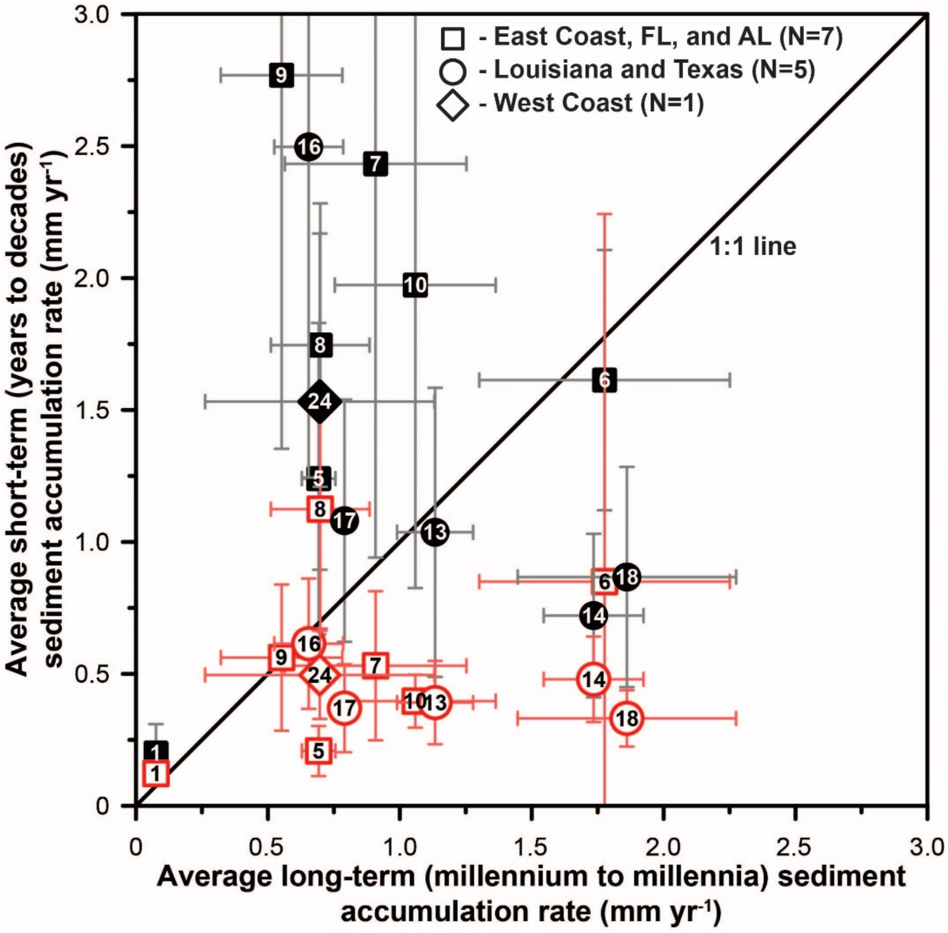


**Supplementary Figure 1: Short-term versus long-term sediment accumulation rates (SAR).** Average SAR measured over millennium time scales are generally greater than average Pb-210 derived SAR values older than 1890 CE (red symbols) and less than average $^{210}$Pb-derived SAR values older than 1950 CE (black symbols; Supplementary Table 1). The increase in sea-level rise to modern rates and associated increase in sediment accommodation that occurred between 1880 and 1930 CE [24, 25] contributed to this difference. The older $^{210}$Pb-dated intervals of the cores measuring SAR over decadal time scales, is like SAR measured over millennium time scales, indicating that periods of erosion and nondeposition at these environmental settings were similar across two orders of magnitude difference in time scales. If the Sadler effect[26] was influencing the records included in this study, then we would expect SAR to be inversely related to the time scale over which it was determined, but this is not the case. Long-term SAR is based on radiocarbon-dated material with midpoints ages ranging between 660 and 8,325 (median 4,155) Cal yr. BP at different depths at each site. Average long-term SAR is calculated using the depth and date of the radiocarbon sample and the depth and date of the base of the $^{210}$Pb profile, averaged for sites with multiple radiocarbon dates. See Supplementary Data 3 for details on the radiocarbon dates and Supplementary Data 1 and Supplementary Data 2 for details on the $^{210}$Pb profiles. Errors are one s.d. of the mean but are based on measurement errors when only one value was available. Numbers identify the sites as shown in Supplementary Table 1.

**Supplementary Table 1: Long-term (millennium to millennia) and short-term (years to decades) sediment accumulation rates (SAR).**

| **Site number and name** | **Long-term SAR (mean; cm/yr)** | **Long-term SAR (± s.d.)** | **Pre-1950 CE short-term SAR (mean; cm/yr)** | **Pre-1950 CE short-term SAR (± s.d.)** | **Pre-1890 CE short-term SAR (mean; cm/yr)** | **Pre-1890 CE short-term SAR (± s.d.)** |
|---|---|---|---|---|---|---|
| 1. Nastapoka Sound, Hudson Bay, Canada | 0.0076 | 0.0018 | 0.0200 | 0.0110 | 0.0122 | 0.0005 |
| 5. Pocomoke Sound, Chesapeake Bay, VA, USA | 0.0692 | 0.0064 | 0.1240 | 0.0590 | 0.0208 | 0.0095 |
| 6. Albemarle Estuarine System, NC, USA | 0.1776 | 0.0475 | 0.1613 | 0.0493 | 0.0850 | 0.1393 |
| 7. Pamlico River Estuary, NC, USA | 0.0908 | 0.0344 | 0.2433 | 0.1492 | 0.0531 | 0.0282 |
| 8. Neuse River Estuary, NC, USA | 0.0698 | 0.0187 | 0.1746 | 0.0537 | 0.1124 | 0.0452 |
| 9. Florida Bay, FL, USA | 0.0552 | 0.0230 | 0.2769 | 0.1416 | 0.0562 | 0.0277 |
| 10. Mobile Bay, AL, USA | 0.1059 | 0.0305 | 0.1973 | 0.1148 | 0.0397 | 0.0100 |
| 13. Barataria Bay, LA, USA | 0.1134 | 0.0144* | 0.1037 | 0.0548 | 0.0391 | 0.0158 |
| 14. Terrebonne Bay, LA, USA | 0.1735 | 0.0189* | 0.0721 | 0.0310 | 0.0479 | 0.0162 |
| 16. Sabine, TX, USA | 0.0655 | 0.0131 | 0.2497 | 0.1253 | 0.0614 | 0.0247 |
| 17. Galveston Bay, TX, USA | 0.0790 | 0.0040 | 0.1081 | 0.0459 | 0.0370 | 0.0167 |
| 18. Corpus Christi Bay, TX, USA | 0.1861 | 0.0414 | 0.0867 | 0.0418 | 0.0331 | 0.0107 |
| 19. San Francisco, CA, USA | 0.0697 | 0.0435 | 0.1532 | 0.0637 | 0.0496 | 0.0167 |

* One observation. Measurement error indicated. See Supplemental Data 3 for details.

## Supplementary References


1 Jolivel, M., Allard, M., St-Onge, G. & Lian, O. Climate change and recent sedimentation in Nastapoka Sound, eastern coast of Hudson Bay. *Canadian Journal of Earth Sciences* 52, 322-337, doi:10.1139/cjes-2014-0132 (2015).

2 Lima, A. L. *et al.* High-resolution historical records from Pettaquamscutt River basin sediments: 1. 210Pb and varve chronologies validate record of 137Cs released by the Chernobyl accident. *Geochimica et Cosmochimica Acta* 69, 1803-1812, doi:10.1016/j.gca.2004.10.009 (2005).

3 Renfro, A. A., Cochran, J. K., Hirschberg, D. J., Bokuniewicz, H. J. & Goodbred, S. L. The sediment budget of an urban coastal lagoon (Jamaica Bay, NY) determined using 234Th and 210Pb. *Estuarine, Coastal and Shelf Science* 180, 136-149, doi:10.1016/j.ecss.2016.06.008 (2016).

4 Benninger, L. K. & Krishnaswami, S. Sedimentary processes in the inner New York Bight: evidence from excess $^{210}$Pb and $^{239,240}$Pu. *Earth and Planetary Science Letters* 53, 158-174 (1981).

5 Holmes, C. W. & Marot, M. Chapter 2. Sediment and chemical flux history in the Pocomoke Sound as defined by short lived isotopic analyses. 23-57 (United States Geological Survey, Reston, 2004).

6 Corbett, D. R., Vance, D., Letrick, E., Mallinson, D. & Culver, S. Decadal-scale sediment dynamics and environmental change in the Albemarle Estuarine System, North Carolina. *Estuarine, Coastal, and Shelf Science* 71, 717-729 (2007).

7 Cooper, S. R., McGlothlin, S. K., Madritch, M. & Jones, D. L. Paleoecological evidence of human impacts on the Neuse and Pamlico Estuaries of North Carolina, USA. *Estuaries* 27, 617-633 (2004).

8 Benninger, L. K. & Wells, J. T. Sources of sediment to the Neuse River estuary, North Carolina. *Marine Chemistry* 43, 137-156 (1993).

9 Wachnicka, A., Gaiser, E. & Collins, L. S. Correspondence of historic salinity fluctuations in Florida Bay, USA, to atmospheric variability and anthropogenic changes. *Journal of Paleolimnology* 49, 103-115, doi:10.1007/s10933-011-9534-9 (2013).

10 Holmes, C.W., Robbins, J.A., Halley, R.B., and Bothner, M. Sediment dynamics of Florida Bay mud banks of a decadal time scale, *Bulletins of American Paleontology*, 361, 30-40 (2001).

11 Orem, W. H., Holmes, C. W., Kendall, C., Lerch, H. E., Bates, A. L., Silva, S. R., and Hedgman, C. Geochemistry of Florida Bay sediments: nutrient history at five sites in eastern and central Florida Bay. *Journal of Coastal Research*, 1055-1071 (1999).

12 Smith, C. G. & Osterman, L. E. An Evaluation of temporal changes in sediment accumulation and impacts on carbon burial in Mobile Bay, Alabama, USA. *Estuaries and Coasts* 37, 1092-1106, doi:10.1007/s12237-013-9731-z (2014).

13 Draut, A. E., Kineke, G. C., Velasco, D. W., Allison, M. A. & Prime, R. J. Influence of the Atchafalaya River on recent evolution of the chenier-plain inner continental shelf,

northern Gulf of Mexico. *Continental Shelf Research* 25, 91-112, doi:10.1016/j.csr.2004.09.002 (2005).

14 Ravichandran, M., Baskaran, M., Santschi, P. H. & Bianchi, T. S. Geochronology of sediments in the Sabine-Neches estuary. *Chemical Geology* 125, 291-306 (1995).

15 Santschi, P. H., Presley, B. J., Wade, T. L., Garcia-Romero, B. & Baskaran, M. Historical contamination of PAHs, PCBs, DDTs and heavy metals in Mississippi River Delta, Galveston Bay and Tampa Bay sediment cores. *Marine Environmental Research* 52, 51-79 (2001).

16 Yeager, K. M., Santschi, P. H., Schindler, K. J., Andres, M. J. & Weaver, E. A. The relative importance of terrestrial versus marine sediment sources to the Nueces-Corpus Christi Estuary, Texas: an isotopic approach. *Estuaries and Coasts* 29, 443-454 (2006).

17 Diaz-Asencio, M. *et al.* One century sedimentary record of Hg and Pb pollution in the Sagua estuary (Cuba) derived from $^{210}$Pb and $^{137}$Cs chronology. *Marine Pollution Bulletin* 59, 108-115, doi:10.1016/j.marpolbul.2009.02.010 (2009).

18 Alonso-Hernandez, C. M. *et al.* Recent changes in sedimentation regime in Cienfuegos Bay, Cuba, as inferred from $^{210}$Pb and $^{137}$Cs vertical profiles. *Continental Shelf Research* 26, 153-167, doi:10.1016/j.csr.2005.08.026 (2006).

19 Ruiz-Fernandez, A. *et al.* Changes of coastal sedimentation in the Gulf of Tehuantepec, South Pacific Mexico, over the last 100 years from short-lived radionuclide measurements. *Estuarine Coastal and Shelf Science* 82, 525-536 (2009).

20 Ruiz-Fernandez, A. C., Hillaire-Marcel, C., Ghaleb, B., Soto-Jimenez, M. & Paez-Osuna, F. Recent sedimentary history of anthropogenic impacts on the Culiacan River Estuary, northwestern Mexico: geochemical evidence from organic matter and nutrients. *Environmental Pollution* 118, 365-377 (2002).

21 Alexander, C. R. & Lee, H. J. in *Earth science in the urban ocean: the Southern California Continental Borderland* Special Paper 454 (eds H.J. Lee & W.R. Normark) 69-87 (Geological Society of America, 2009).

22 Fuller, C. C., van Geen, A., Baskaran, M. & Anima, R. Sediment chronology in San Francisco Bay, California, defined by $^{210}$Pb, $^{234}$Th, $^{137}$Cs, and $^{239,240}$Pu. *Marine Chemistry* 64, 7-27 (1999).

23 Wheatcroft, R. A. & Sommerfield, C. K. River sediment flux and shelf sediment accumulation rates on the Pacific Northwest margin. *Continental Shelf Research* 25, 311-332, doi:10.1016/j.csr.2004.10.001 (2005).

24 Church, J.A., White, N.J., A 20th century acceleration in global sea-level rise. Geophysical Research Letters 33 (2006).

25 Kemp, A. C. et al. Climate related sea-level variations over the past two millennia. Proceedings of the National Academy of Sciences, doi:10.1073/pnas.1015619108 (2011).

26 Sadler, P. M. Sediment Accumulation Rates and the Completeness of Stratigraphic Sections. The Journal of Geology 89, 569-584 (1981).
