# Supplementary material for: Coastal sedimentation across North America doubled in the 20th century despite river dams
Source: Nat Commun. 2020 Jun 26;11:3249. doi: 10.1038/s41467-020-16994-z (PMC7319974; doi:10.1038/s41467-020-16994-z)
Supplement: Supplementary file 3 — Description of Additional Supplementary Files [file 41467_2020_16994_MOESM3_ESM.pdf]

**Description of Additional Supplementary Files**

File Name: Supplementary Data 1
Description: Summary information on cores, sedimentation, coastal population, and sea-level rise. These data are plotted on Figures 1, 2, 5, and 6. Sites are numbered the same as in Figure 1.

File Name: Supplementary Data 2
Description: Mass accumulation rates and sediment accumulation rates. These data are plotted in Figure 3. Sites are numbered the same as in Figure 1. Error refers to measurement error and details are presented in Methods.

File Name: Supplementary Data 3
Description: Long-term and short-term sediment accumulation rates. Data averages are presented in Supplemental Table 1. Long term rates are based on radiocarbon dates.
